# Quantifying the Digital Ecosystem of Commercial Technologies for Decentralized Clinical Trial Operations: Scoping Review

**DOI:** 10.2196/86011

**Published:** 2026-11-12

**Authors:** Lampros Mpaltadoros, Simone Huber, Thanos G Stavropoulos, Bianca Buchgraber-Schnalzer, Jaime Fons-Martínez, Carlos Murciano-Gamborino, Baptiste Alcalde, Lina Pérez-Breva, Spiros Nikolopoulos, Ioannis Kompatsiaris, Sten Hanke

**Affiliations:** 1 Information Technologies Institute, Centre for Research and Technology Hellas, 6th Km Charilaou-Thermi, Thessaloniki, 57001, Greece, +306973900849; 2 Institute of eHealth, FH JOANNEUM GmbH – University of Applied Sciences, Graz, Austria; 3 Vaccines Research Department, Fundación para el Fomento de la Investigación Sanitaria y Biomédica de la Comunitat Valenciana, Valencia, Spain; 4 BBMRI-ERIC, Graz, Austria

**Keywords:** decentralized clinical trials, scoping review, clinical trial operations, clinical trial design, patient recruitment, data acquisition, data processing, patient engagement, intervention, reporting, digital health technologies

## Abstract

**Background:**

Decentralized clinical trials (DCTs) are reshaping clinical research through digital technologies such as telemedicine, mobile apps, wearable and sensor-based tools, and digital platforms. These technologies can support participation, safety, accessibility, data collection, monitoring, and trial operations. Although DCT adoption accelerated during the COVID-19 pandemic, the commercial technology landscape remains fragmented and continues to evolve rapidly. There is limited understanding of how commercial technologies align with operational activities across the DCT life cycle. The Basic Building Block (BBB) framework used in this review divides the DCT life cycle into 7 functional areas: setup and design; recruitment and enrollment; data acquisition and processing; operation and coordination; patient engagement; intervention and follow-up; and close-out and reporting.

**Objective:**

This scoping review aimed to map commercial technologies supporting DCT operations and assess their availability and functional alignment with clinical trial processes.

**Methods:**

A scoping review of commercial technologies was conducted from January 2024 to June 2025 to update a previous 2020 technology scan, with a search rerun in June 2026 for update and verification purposes. Eligible technologies were commercially available, publicly documented, and relevant to at least 1 BBB. The review was reported in accordance with PRISMA-ScR (Preferred Reporting Items for Systematic Reviews and Meta-Analyses Extension for Scoping Reviews), with search reporting guided by PRISMA-S (Preferred Reporting Items for Systematic Reviews and Meta-Analyses Literature Search extension) where applicable. Seven 2-person reviewer groups reassessed previously identified technologies and identified additional technologies by analyzing publicly available English-language sources, including vendor and company websites, product documentation, public reports, case studies, company announcements, and relevant academic literature. Technologies were categorized by BBB coverage, classified as single-BBB if they supported 1 BBB and multiple-BBB if they supported more than 1 BBB, and analyzed using a co-occurrence matrix.

**Results:**

The final catalog included 329 commercial technologies, spanning the DCT life cycle. Most were multiple-BBB technologies, with 293 multiple-BBB and 36 single-BBB technologies. Operation and coordination was the most frequently covered BBB (n=220), followed by data acquisition and processing (n=212), setup and design (n=189), patient engagement (n=165), close-out and reporting (n=125), intervention and follow-up (n=122), and recruitment and enrollment (n=120). The strongest co-occurrence pairings were data acquisition and processing with operation and coordination; setup and design with operation and coordination; and setup and design with data acquisition and processing. The weakest pairings involved close-out and reporting with intervention and follow-up, with recruitment and enrollment, and with patient engagement.

**Conclusions:**

This scoping review provides an updated structured mapping of 329 commercial technologies supporting DCT operations. By combining a commercial technology scan with the BBB framework, the review quantifies functional coverage and co-occurrence across the DCT life cycle. The findings can support technology selection for decentralized and hybrid trials while highlighting operational areas where technology coverage seems to be less commonly combined.

## Introduction

A decentralized clinical trial (DCT) is defined as “a clinical trial where some or all of the trial-related activities occur at locations other than traditional clinical trial sites” [1]. This means that participants can take part in a study from home or through local health care facilities [2], in contrast to traditional clinic settings. There has been a rise in elements and activities related to DCTs [3], where adoption is steadily increasing. Particularly, due to the COVID-19 pandemic, there was a further acceleration of DCT adoption, as there was a pressing need to minimize infection risks, delays, and disruptions [4].

DCTs rely on digital technologies across clinical trial activities to support participation, safety, accessibility, data collection, monitoring, and trial operations [5]. Key technologies include telemedicine technologies for recruitment, informed consent, assessment, monitoring, and engagement [6]; mobile technologies and mHealth apps for participant engagement and data collection [5,7]; and wearable, sensor-based, and Internet of Things technologies that can capture physiological and environmental data depending on the trial design [8-12].

In addition to technologies, several large-scale initiatives have emerged to support the broader adoption of technologies in clinical trials and DCTs. The Innovative Health Initiative [13] is a public-private partnership in Europe that funds collaborative research across academia, industry, and regulators. One such project, funded under Innovative Medicines Initiative 2, is Trials@Home [14], which aimed to reshape clinical trial design, conduct, and operations by developing recommendations for DCTs in Europe. In this context, a fundamental objective was to scan for existing technologies suitable for DCT operations and select appropriate combinations for use in RADIAL, a pan-EU clinical trial pilot. This created a need for a structured overview of the commercial technology landscape available to support decentralized trial conduct.

Strategic selection of technologies is necessary to ensure all trial processes are covered while navigating the complex operational and regulatory landscape of DCTs, particularly in light of disruptions such as COVID-19 [15]. In DCTs, technology is applied across the trial life cycle, from recruitment and enrollment to informed consent, interventions, patient-reported outcomes, and safety monitoring [16].

Several scoping reviews have examined digital technologies used in clinical trials and clinical research. Harrison et al [17] conducted a formative feasibility scoping review for digital technologies in registered clinical trials. Koller et al [18] reviewed digital therapeutics and software as a medical device in decentralized clinical trials. Additional scoping reviews focused on specific clinical trial operations, including electronic recruitment support systems and AI applications in clinical trials [19,20]. However, these studies focus more on digital technologies in use, specific technology types, or individual trial operations rather than providing a structured mapping of technologies across the full DCT operational life cycle. They are also more research-oriented and do not map the commercial landscape. A gap emerges in understanding how the available commercial technologies cover the clinical trial operations, whether they support single or multiple phases, and how their functional coverage might co-occur within available technologies.

To identify and classify commercial technologies consistently, the review first required a structured way to represent the main operational phases of clinical trials and to map each technology to the activities it supports. Clinical trial operations have been conceptualized in different ways across the literature. The following fundamental operations have been suggested [21]: Trial Design and Registration, Participant Enrollment and Execution, Study Completion and Analysis, and Regulatory and Publication activities. In another case, the clinical trial life cycle has been described as progressing from Trial Design and Planning through Trial Conduct and Operations (Recruitment, Enrollment, Participation), followed by Dissemination, Communication, and Post-approval activities [22]. IT supports processes by involving multiple systems: Clinical Trial Management System, patient recruitment support system, digital consent/eConsent solution, Identity and Pseudonymization services, and visit scheduling/task and workflow management tool.

To support consistent classification of commercial technologies, this review uses the Basic Building Block (BBB) framework to segment clinical trial operations into functional areas relevant to decentralized and digitally enabled trial conduct. The BBB framework was developed within the Trials@Home RADIAL pan-EU study and was first applied in the 2020 technology scan [23]. Its development involved defining key operational activities, refining them through specialized workgroups, and validating them through a consensus-based process. The framework was then used to structure a market scan of technologies and map identified technologies to vendors and supported trial activities.

Building on this foundation, the present study applies the BBB framework to segment DCTs into distinct operational phases. Each BBB encompasses specific activities and associated data flows, supporting the classification of appropriate technologies. This modular, activity-based structure enables flexible and interoperable system design across the clinical trial life cycle. The 7 BBBs are organized as follows:

- Setup and design: Phase in which clinical trial planning, feasibility, and infrastructure are prepared, submitted, and designed to allow successful trial execution.
- Recruitment and enrollment: Phase in which potential participants are identified, assessed for eligibility, and enrolled in the trial.
- Data acquisition and processing: (1) Collection, storage, and management of study data (eg, participant, device, and sample data), ensuring compliance with protocol and Good Clinical Practice, and (2) data verification, transformation, and analysis.
- Operation and coordination: Processes relating to the operational conduct of the study.
- Patient engagement: Activities to support and maintain participant engagement throughout the trial.
- Intervention and follow-up: Activities that collect study data and/or deliver interventions in accordance with the study protocol.
- Close-out and reporting: Activities to conclude the trial, ensuring all processes are completed, materials are accounted for and decommissioned, and results are appropriately reported and published.

Figure 1 shows how the BBB framework aligns with the main phases of the clinical trial life cycle. Table 1 summarizes the activities mapped to each BBB, which were later used to structure and categorize available technologies.

**Figure 1.**
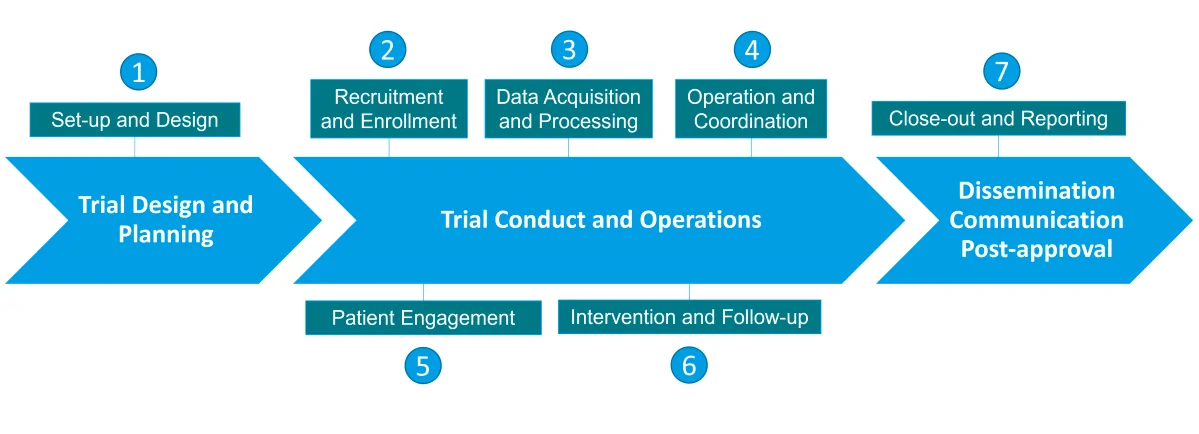
Mapping of basic building blocks (BBBs) onto the clinical trial life cycle (as described in Pung and Rienhoff [22]).

**Table 1.** Basic building blocks (BBBs) and related activities.

Basic building block	Activities
Setup and design	Protocol development; operational feasibility assessment; trial registration; informed consent form development; study branding; participant and site education strategy; site startup; ethics and regulatory approvals; technology setup; investigational medical product (IMP) supply; operational setup
Recruitment and enrollment	Participant outreach and prescreening; participant education; informed consent; screening and randomization; IMP supply; patient technology enablement
Data acquisition and processing	Phase 1: Study data management; data collection middleware; real-world data capture and management; clinical data repository management Phase 2: eCRF^a^ and system query design; data reconciliation and query management; database lock; data transformation, standardization, and analysis
Operation and coordination	Clinical and performance monitoring; inspection and system approval facilitation; documentation management; home health and telemedicine visit management; regulatory and vendor management; safety data management; operational analytics; protocol and GCP^b^ deviation management; study oversight
Patient engagement	Patient engagement planning; social listening and patient landscape analysis; patient advocacy mapping; participant communication and updates; recruitment and retention incentives; concierge services or travel reimbursement; behavioral incentives; patient satisfaction surveys; advisory board engagement; patient-HCP^c^ interaction; direct messaging; patient community development
Intervention and follow-up	Self-intervention and monitoring; home health visits; telemedicine visits
Close-out and reporting	Decommissioning; archiving; study report production; clinical and operational results publication; scientific dissemination

a eCRF: electronic case report form.

b GCP: Good Clinical Practice.

c HCP: health care professional.

Building on the previous 2020 technology scan [23], the aim of this paper is to update and extend that earlier work by mapping commercially available technologies that currently support decentralized and digitally enabled clinical trial operations. Specifically, this review reassesses previously identified technologies, identifies additional eligible technologies, and evaluates their current availability and functional alignment with the BBB framework across the DCT life cycle. This update was necessary because the digital health and clinical-trial technology landscape continues to evolve rapidly with new trends and technological advancements [24], particularly following the disruptive impact of the COVID-19 pandemic on trial conduct. By providing an updated catalog of technologies, quantifying their BBB coverage, and identifying patterns of overall and co-occurring functional coverage, this review aims to support more strategic and informed technology selection for DCT implementation. The review was guided by the following question: which commercial technologies are currently available to support DCT operations, and how do they align with and co-occur across the BBB framework?

## Methods

### Protocol and Registration

This study was conducted as a scoping review to map the landscape of commercial technologies supporting DCTs. The review was designed to update and extend a previous 2020 technology scan [23] by reassessing previously identified technologies, identifying additional technologies, and providing an up-to-date overview of available technologies across the DCT life cycle.

In this review, the units of selection and charting were commercial technologies, while publicly available documents and web sources were used as evidence to verify availability, functionality, and BBB relevance.

The review was reported in accordance with the PRISMA-ScR (Preferred Reporting Items for Systematic Reviews and Meta-Analyses Extension for Scoping Reviews) [25]. Search reporting was additionally guided by PRISMA-S (Preferred Reporting Items for Systematic Reviews and Meta-Analyses Literature Search Extension) [26] where applicable, with adaptations for a web-based commercial technology landscape review. No protocol for this scoping review was registered. The completed PRISMA-ScR and PRISMA-S checklists are provided in Checklists 1 and 2.

The overall workflow of the scoping review and technology scan is summarized in Textbox 1. Each stage is described in detail in the following Methods subsections.

Textbox 1. **High-level overview of the scoping review methodology.**

1. Protocol and registration

- Scoping review of commercial decentralized clinical trial technologies
- Update of 2020 technology scan
- PRISMA-ScR (Preferred Reporting Items for Systematic Reviews and Meta-Analyses Extension for Scoping Reviews) reported
- PRISMA-S (Preferred Reporting Items for Systematic Reviews and Meta-Analyses Literature Search Extension)–guided search reporting

2. Eligibility criteria

- Commercial technologies supporting 1 or more basic building blocks (BBBs)
- Sufficient public documentation required
- Unavailable, duplicate, or irrelevant technologies excluded

3. Information sources

- Public English-language sources
- Vendor/product websites and documentation
- Reports, case studies, announcements, and relevant literature

4. Search

- BBB-aligned web-based search terms
- January 2024 to June 2025 structured update
- June 2026 search rerun and verification
- Technology-specific verification searches

5. Selection of sources of evidence

- Existing technologies reassessed; new technologies screened
- Assigned reviewer and second-reviewer check

- Status assigned: active, updated, removed, or new

6. Data-charting process

- Structured 2026 review fields completed
- AI-assisted draft suggestions used during the June 2026 verification
- Evidence and BBB coding checked by reviewers

7. Data items

- Technology name, vendor, link, source, and date checked
- BBB YES/NO coverage recorded
- Single-/multiple-BBB classification calculated and reviewer notes

8. Critical appraisal

- Formal critical appraisal not conducted
- Effectiveness and risk of bias out of scope

9. Synthesis of results

- Descriptive BBB coverage statistics
- Co-occurrence matrix and heatmap
- Final catalog of 329 technologies
- Multimedia Appendix 1 dataset

### Eligibility Criteria

Technologies were included if they met all of the following criteria:

- They represented commercially available digital technologies applicable to clinical trial operations.
- They supported activities related to at least 1 BBB.
- They had sufficient publicly available documentation to allow classification.

For the purposes of this study, “sufficient publicly available documentation” was defined as the presence of at least one of the following: a detailed technology description on an official vendor website; technical documentation, white papers, or product brochures; or case studies or implementation descriptions explicitly describing functionality. Technologies were required to provide enough detail to allow mapping to at least 1 BBB activity.

The review included publicly accessible English-language sources identified during the January 2024 to June 2025 structured update and the June 2026 search rerun and verification step. Commercial digital health, clinical trial, remote monitoring, patient engagement, data capture, trial management, and related technology platforms were eligible when their publicly available documentation supported relevance to clinical trial operations.

Technologies were excluded if they met any of the following criteria:

- They were no longer actively maintained or commercially available at the time of the relevant search or verification step.
- They represented duplicate entries from multiple sources or reviewer groups.
- They lacked sufficient detail to support classification against at least 1 BBB activity.
- They were not sufficiently related to clinical trial operations.

When information was incomplete or ambiguous, inclusion decisions were made conservatively. Technologies were included only when the available public information provided reasonable confidence that the technology was relevant to clinical trial operations and could be mapped to at least 1 BBB based on explicit statements in the source material. If this could not be clearly determined, the technology was excluded to reduce the risk of misclassification.

### Information Sources

The review used a staged evidence-identification process. The starting point was the previous Trials@Home technology scan conducted in 2020, which served as the baseline dataset of commercial technologies for reassessment [23]. A structured scoping review update was then conducted from January 2024 to June 2025 to reassess previously identified technologies and identify additional commercial technologies relevant to decentralized clinical trial operations.

The most recent update and verification step was completed in June 2026. This rerun used the same BBB-aligned search strategy and was used to verify the current status of previously identified technologies and identify additional eligible technologies.

Information sources included publicly accessible English-language sources. These included official vendor and company websites, product pages, product documentation, technical documentation, product brochures, white papers, publicly available reports, case studies or implementation descriptions, press releases or company announcements where relevant, and relevant academic literature. The search was global in scope.

Paywalled sources, nonpublic vendor-specific procurement data, pricing information, commercial intelligence databases, and sources requiring direct vendor engagement were not systematically searched. Study registries and bibliographic databases were not used as the primary search route because the objective was to map commercially available technologies rather than clinical studies. However, relevant academic literature and prior work were used where applicable to contextualize the review and support the conceptual framework, but they were not the primary route for identifying commercial technologies.

### Search

The search was conducted in 2 phases. First, a structured scoping review update was conducted from January 2024 to June 2025. During this phase, reviewer pairs used BBB-aligned search terms to reassess technologies identified in the 2020 scan and identify additional commercial technologies. Second, the search was rerun in June 2026 as an update and verification step using the same BBB-aligned search approach. The June 2026 rerun was used to ensure that the catalog was as up to date as possible, verify the status of previously identified technologies, and identify newly eligible technologies.

Searches were organized by BBB domain and source category. The web-based source categories included official vendor and product pages, vendor documentation and help resources, official service-provider websites, app-store listings and software directories, official institutional or registry websites, publicly available reports, case studies or implementation descriptions, press releases or acquisition announcements, professional or company profile sources, and relevant academic literature where applicable. In cases where 1 source was insufficient to classify a technology, multiple public sources were used to confirm technology status, vendor ownership, functionality, and BBB relevance.

For each BBB, search terms combined clinical trial, decentralized clinical trial, remote trial, hybrid trial, or digital trial terms with terms describing relevant technology functions. Because the search was web-based and focused on commercial technologies rather than bibliographic records, these search terms were used to guide structured and iterative searches across public web sources rather than as fixed database search strings. When a potentially eligible technology was identified, additional technology-specific searches were performed using current and previous technology names, vendor names, and known product names to verify current availability, ownership, links, public documentation, and relevance to one or more BBBs. During the June 2026 rerun, this technology-specific verification was repeated for previously identified technologies.

The BBB-aligned search terms used to guide the web-based commercial technology search are presented in Table 2. These search terms were used during the January 2024 to June 2025 structured update and the June 2026 rerun. No published search filters were used.

**Table 2.** Basic building block (BBB)–aligned search terms used to guide the web-based commercial technology search.

Basic building block	Search terms used
Setup and design	“clinical trial design software”; “protocol design tool” AND “clinical trial”; “trial feasibility platform” AND “clinical trial”; “study startup software” AND “clinical trial”; “clinical trial planning platform”; “decentralized clinical trial” AND “trial design software”; “hybrid clinical trial” AND “study startup”
Recruitment and enrollment	“patient recruitment platform” AND “clinical trial”; “clinical trial matching platform”; “clinical trial recruitment software”; “digital consent” AND “clinical trial”; “eConsent” AND “clinical trial”; “decentralized clinical trial” AND “patient recruitment”; “remote clinical trial” AND “enrollment”
Data acquisition and processing	“clinical data management system”; “eCRF” AND “clinical trial”; “electronic data capture” AND “clinical trial”; “remote data collection” AND “clinical trial”; “wearable data platform” AND “clinical trial”; “digital biomarker platform” AND “clinical trial”; “decentralized clinical trial” AND “data capture”
Operation and coordination	“clinical trial management system”; “CTMS” AND “clinical trial”; “trial monitoring software”; “remote monitoring” AND “clinical trial”; “site management platform” AND “clinical trial”; “decentralized clinical trial” AND “trial operations”; “clinical trial oversight platform”
Patient engagement	“patient engagement platform” AND “clinical trial”; “participant engagement” AND “clinical trial”; “clinical trial retention platform”; “patient app” AND “clinical trial”; “participant communication platform” AND “clinical trial”; “decentralized clinical trial” AND “patient engagement”; “remote clinical trial” AND “participant retention”
Intervention and follow-up	“telemedicine” AND “clinical trial”; “remote visit platform” AND “clinical trial”; “virtual visit” AND “clinical trial”; “home health” AND “clinical trial”; “remote follow-up” AND “clinical trial”; “decentralized clinical trial” AND “telemedicine”; “hybrid clinical trial” AND “remote visits”
Close-out and reporting	“clinical trial reporting software”; “study report production” AND “clinical trial”; “clinical trial closeout software”; “regulatory submission software” AND “clinical trial”; “trial archiving software”; “clinical trial document management” AND “closeout”; “clinical trial results reporting platform”

Because the review focused on commercial technologies rather than bibliographic records, searches were conducted primarily through web-based searches and source-specific searches of public vendor, product, documentation, registry, software directory, institutional, and publication sources. The search was limited to publicly available English-language information. No date, geography, study-design, or publication-type limits were applied beyond the requirement that technologies were commercially available and sufficiently documented at the time of review. The most recent search rerun and verification step was completed in June 2026.

### Selection of Sources of Evidence

For the selection of sources of evidence, reviewers with expertise in clinical research and health care technologies were organized into 7 two-person groups, with each group assigned to 1 BBB. Reviewer pairs reassessed technologies from the 2020 baseline scan and screened newly identified technologies from the January 2024 to June 2025 structured update.

For both existing catalog entries and newly identified candidate technologies, the selection process involved reviewer-based verification. Existing technologies from the 2020 baseline scan were reassessed, while newly identified technologies were screened against the same eligibility criteria. Each entry was reviewed by an assigned reviewer and then checked by a second reviewer before final acceptance, update, exclusion, or referral for further discussion.

Technologies were screened against the eligibility criteria and classified as active, updated, removed, or new. Active technologies were defined as technologies that remained active and covered the same activities as previously recorded. Updated technologies were technologies that remained active but had expanded, reduced, or otherwise changed functionality, requiring an updated description or BBB classification. Removed technologies were technologies that no longer existed, could not be found, were no longer commercially available, or no longer met the eligibility criteria. New technologies were newly identified technologies that met the eligibility criteria and were added to the dataset.

The June 2026 search rerun and verification step followed the same selection approach. Previously identified technologies were rechecked for current availability, vendor information, public documentation, links, functionality, and BBB relevance. Newly identified technologies encountered during the rerun were screened against the same eligibility criteria.

Entries with incomplete or inconsistent information, nonfunctional or inaccessible technologies, unclear relevance to clinical trial operations, or insufficient publicly available documentation were removed during data cleaning. Duplicate entries were removed manually through cross-checking reviewer outputs and source lists. No specialized deduplication software was used. All technologies classified as active, updated, or new after final cleaning were included in the analyses.

### Data-Charting Process

A structured data-charting form was used to extract and update information for each eligible technology. Data charting was performed by expert reviewer pairs assigned to each BBB. Reviewers extracted information from publicly available sources and recorded the current technology name, vendor, best available link, evidence source, review status, supported activities, and BBB coverage.

During the June 2026 supplementary search rerun and verification step, both existing catalog entries and newly identified candidate technologies were processed through a structured, reviewer-verified workflow. Existing entries were reassessed using the information already available in the catalog, while newly identified candidate technologies were charted using the same 2026 review fields. These fields included review status, action recommendation, current technology name, vendor, current link, evidence source, updated BBB coding, revised supported activities, and reviewer notes.

During the June 2026 catalog update and verification step, generative AI with web-search capability was used under human supervision as an assistive tool to generate structured draft suggestions for human reviewer verification. It was not used to conduct the original January 2024 to June 2025 structured review.

For the June 2026 update, a standardized prompt was used to generate suggestions based on publicly available information. These suggestions included public-evidence summaries, possible updates to technology and vendor information, current public links, supported-activity descriptions, and preliminary BBB coding suggestions. The standardized prompt used to support catalog verification is provided in Multimedia Appendix 2. The prompt included additional internal audit-trail and reviewer-workflow fields to support verification, but only the final public catalog fields were retained in Multimedia Appendix 1.

All AI-assisted outputs were treated only as suggestions and draft material. The actual catalog review process was conducted by 2 human reviewers, who verified the public evidence, reviewed technology and vendor information, assessed supported activities, and finalized BBB classifications before final acceptance, update, exclusion, or referral for team discussion. Generative AI outputs were not treated as evidence sources and did not determine inclusion, exclusion, or final BBB classification.

Technologies were categorized based on their coverage of the BBBs using the established BBB definitions described above. The classification process was conducted by reviewer pairs to enhance consistency and reduce individual reviewer bias. In cases of disagreement, classifications were discussed between reviewers until consensus was reached. Where necessary, an additional review was undertaken by a third expert to ensure alignment with the BBB definitions. Formal interrater agreement statistics were not calculated, as final classifications were reached through expert consensus within BBB-specific reviewer pairs, with third-reviewer input where needed.

The charting form was updated during both the January 2024 to June 2025 structured scoping review update and the June 2026 supplementary search rerun and verification step. During the June 2026 rerun, previously charted entries were rechecked and updated where changes in technology name, vendor, availability, evidence source, functionality, or BBB classification were identified.

### Data Items

The following data items were charted for each technology: final 2026 verification status, technology name, vendor, best available public link, public evidence source used, date checked, BBB coverage, number of BBBs covered, single-BBB or multiple-BBB classification, and publicly reported supported activities.

BBB coverage was recorded using binary “YES” or “NO” values for each of the 7 BBBs: setup and design; recruitment and enrollment; data acquisition and processing; operation and coordination; patient engagement; intervention and follow-up; and close-out and reporting. A “YES” value indicated that publicly available evidence supported the technology’s relevance to that BBB. A “NO” value indicated that the available evidence did not support classification under that BBB. Additional quality-control fields were used during the 2026 update, including reviewer initials, second reviewer initials, second reviewer check, needs team discussion, and team discussion topic.

Technologies were classified as single-BBB technologies when they covered only one BBB and as multiple-BBB technologies when they covered more than 1 BBB. The total number of BBBs covered by each technology was calculated by summing the number of “YES” values across the 7 BBB columns. This provided a measure of each technology’s breadth of functional coverage across the DCT life cycle.

### Critical Appraisal of Individual Sources of Evidence

This scoping review aimed to map the commercial landscape of technologies supporting decentralized clinical trial operations and classify available technologies according to their functional alignment with the BBB framework. Critical appraisal is typically used to assess the methodological quality, reliability, or risk of bias of individual evidence sources; however, this review did not aim to evaluate the effectiveness, clinical performance, or quality of individual technologies or studies. Therefore, a formal critical appraisal was not conducted.

### Synthesis of Results

The final charted dataset was synthesized descriptively. Technologies were summarized by review status, BBB coverage, number of BBBs covered, and single-BBB or multiple-BBB classification. Counts were calculated for the total number of technologies supporting each BBB, the number of single-BBB and multiple-BBB technologies, and the distribution of technologies by number of BBBs covered.

To examine relationships between BBBs, a co-occurrence analysis was conducted using a binary matrix representation, a method widely applied in information science [27]. The structured dataset was converted into a binary matrix, with each row representing 1 technology and each column representing 1 BBB. BBB coverage was coded as 1 when a technology supported a BBB and 0 otherwise.

The co-occurrence matrix was computed by multiplying the transpose of the binary matrix by the matrix itself:


$$C=M^{T}\cdot M$$

In this matrix, each off-diagonal value represented the number of technologies supporting both BBBs in a pair, while diagonal values represented the total number of technologies supporting each individual BBB. The resulting co-occurrence matrix was visualized as a heatmap to identify strong pairings and underrepresented combinations of BBBs. The analysis was performed in Python 3 [28], using Pandas [29] for matrix encoding and transformation and Seaborn [30] for visualization.

## Results

### Selection of Sources of Evidence

During the January 2024 to June 2025 structured scoping review update, previously identified technologies from the 2020 scan [23] were reassessed and additional technologies were identified. Initially, 473 technologies were assessed. Of these, 83 were removed because they could not be found, had limited information, were no longer relevant, or no longer met the eligibility criteria. This resulted in 390 technologies classified as active, updated, or new.

Subsequent data cleaning was performed to remove incomplete, inconsistent, or otherwise invalid entries. An additional 12 technologies were removed as bad/invalid entries, and 66 duplicates were removed manually through cross-checking reviewer outputs and source lists. This resulted in 312 technologies retained after the primary January 2024 to June 2025 update. These 312 technologies comprised 147 active, 120 updated, and 45 new technologies after the primary update.

The search was then rerun in June 2026 as an update and verification step using the same BBB-aligned strategy. During this rerun, previously retained technologies were rechecked and updated where needed, 25 additional eligible technologies were identified, and 8 previously retained entries were removed or no longer retained after verification and cleaning. The final updated catalog therefore included 329 commercial technologies. By final 2026 verification status, the catalog comprised 248 (75.4%) updated technologies, 56 (17%) active technologies, and 25 (7.6%) new technologies.

The PRISMA-ScR [25] informed flowchart of technology identification, verification, and inclusion process is presented in Figure 2.

**Figure 2.**
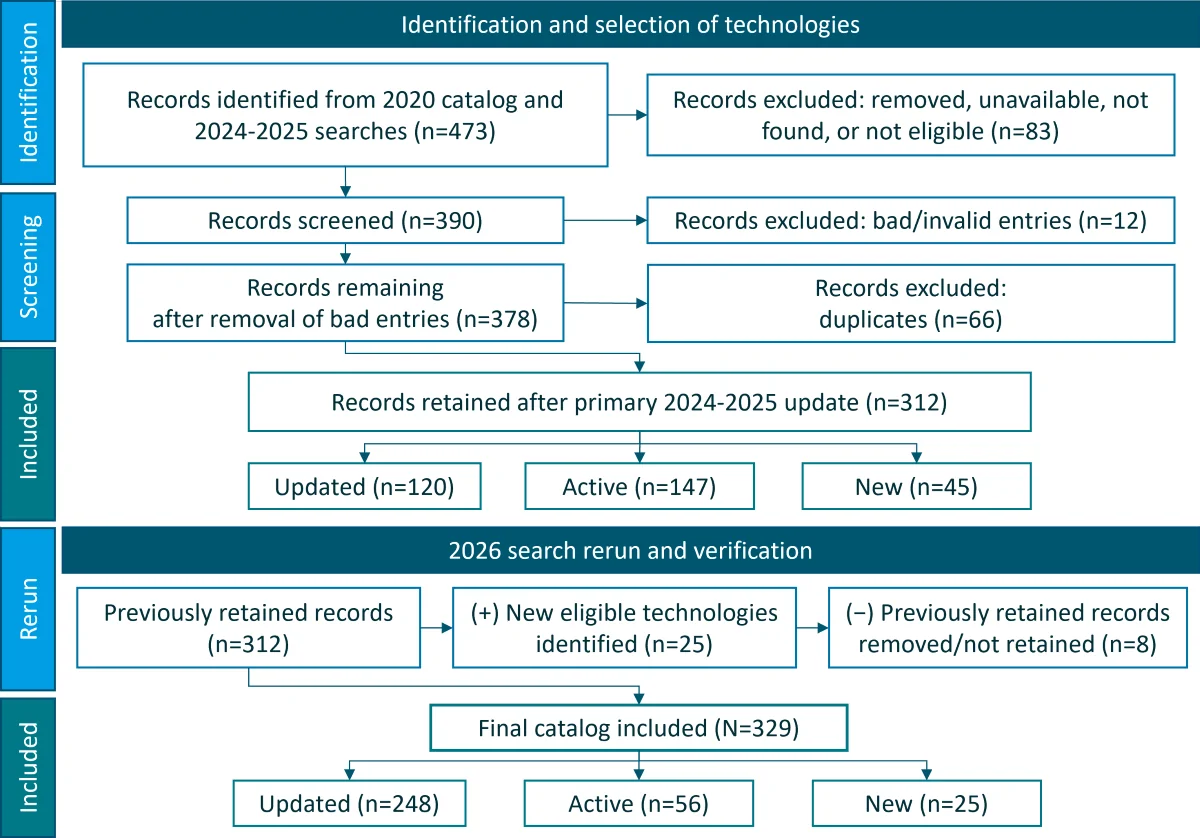
PRISMA-ScR (Preferred Reporting Items for Systematic Reviews and Meta-Analyses Extension for Scoping Reviews)–informed flowchart of technology identification, verification, and inclusion.

The 45 technologies classified as new after the primary 2024-2025 update were new at that stage. Technologies that remained eligible in 2026 were reassessed as active or updated. In the final catalog, new refers only to the 25 technologies first identified during the June 2026 rerun.

### Characteristics of Sources of Evidence

The final catalog included 329 commercial technologies supporting one or more areas of decentralized or digitally enabled clinical trial operations. The technologies were mapped to the 7 BBBs: setup and design; recruitment and enrollment; data acquisition and processing; operation and coordination; patient engagement; intervention and follow-up; and close-out and reporting. Most technologies in the final catalog were classified as multiple-BBB technologies. Overall, 293 of 329 (89.1%) technologies were classified as multiple-BBB technologies, while 36 (10.9%) were classified as single-BBB technologies. The distribution of technologies by number of BBBs covered is presented in Figure 3. In total, 36 technologies covered 1 BBB, 54 covered 2 BBBs, 92 covered 3 BBBs, 68 covered 4 BBBs, 34 covered 5 BBBs, 24 covered 6 BBBs, and 21 covered all 7 BBBs. Charted characteristics for included technology are reported in Multimedia Appendix 1.

**Figure 3.**
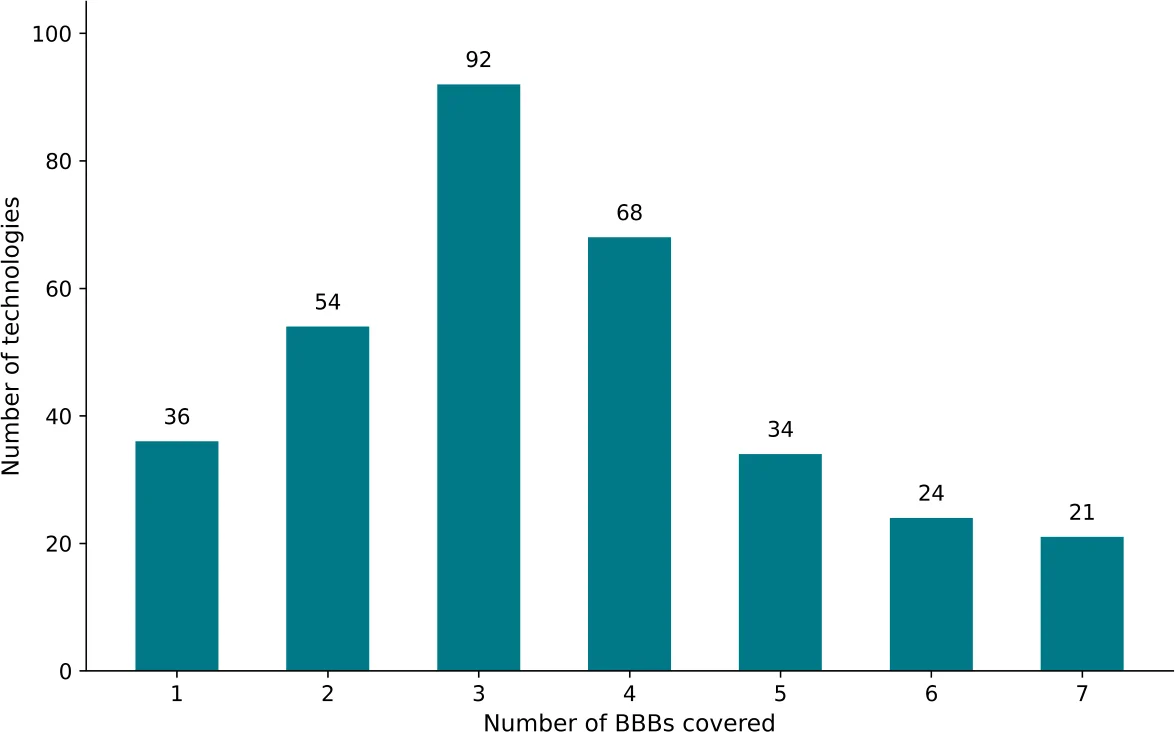
Distribution of technologies by number of basic building blocks (BBBs) covered.

### Results of Individual Sources of Evidence

The BBB categorization of individual technologies is available in Multimedia Appendix 1. This appendix serves as the full charted dataset for the review and includes 1 row per included commercial technology. For each technology, the appendix reports the final 2026 verification status, technology name, vendor, best available public link, public evidence source used, and date checked.

The appendix also reports binary YES/NO coding for each of the 7 BBBs: setup and design; recruitment and enrollment; data acquisition and processing; operation and coordination; patient engagement; intervention and follow-up; and close-out and reporting. Based on this coding, the dataset includes the total number of BBBs covered by each technology and its classification as single-BBB or multiple-BBB. In addition, the appendix provides a description of the publicly reported supported activities.

The appendix therefore provides the technology-level basis for the descriptive BBB coverage statistics, single-BBB and multiple-BBB summaries, and co-occurrence analyses presented in the following section. Only technologies retained after final verification as updated, active, or new were included in the final catalog and subsequent analyses.

### Synthesis of Results

This section synthesizes the technology-level classifications reported in Multimedia Appendix 1 to summarize BBB coverage patterns across the final catalog. Table 3 summarizes the number of technologies covering each BBB across the overall results, single-BBB technologies, and multiple-BBB technologies. Across the full catalog (N=329), operation and coordination was the most frequently covered BBB, with 220 (66.9%) technologies, followed by data acquisition and processing with 212 (64.4%) and setup and design with 189 (57.4%). Patient engagement was covered by 165 (50.2%) technologies, close-out and reporting by 125 (38%), intervention and follow-up by 122 (37.1%), and recruitment and enrollment by 120 (36.5%). Among single-BBB technologies, setup and design was the most frequently represented category with 10 technologies, followed by close-out and reporting with 9, data acquisition and processing with 8, patient engagement with 6, and operation and coordination with 3. No single-BBB technologies were identified for recruitment and enrollment or for intervention and follow-up.

Among multiple-BBB technologies, operation and coordination was the most frequently covered bbb with 217 technologies, followed by data acquisition and processing with 204, setup and design with 179, patient engagement with 159, intervention and follow-up with 122, recruitment and enrollment with 120, and close-out and reporting with 116. Figure 4 presents the single-BBB, multiple-BBB, and overall coverage for each BBB.

**Table 3.** Number of technologies covering each basic building block (BBB) across overall, single-BBB, and multiple-BBB results (N=329).

BBB	Overall results, n	Single-BBB, n	Multiple-BBB, n
Setup and design	189	10	179
Recruitment and enrollment	120	0	120
Data acquisition and processing	212	8	204
Operation and coordination	220	3	217
Patient engagement	165	6	159
Intervention and follow-up	122	0	122
Close-out and reporting	125	9	116

BBB co-occurrence is presented as a co-occurrence matrix in Figure 5, illustrating the extent to which technologies supported multiple BBBs simultaneously. The diagonal values represent the total number of technologies supporting each individual BBB. Off-diagonal values represent the number of technologies supporting both BBBs in a pair. Darker shades indicate stronger co-occurrence, highlighting common pairings and underrepresented combinations across the DCT operational life cycle.

**Figure 4.**
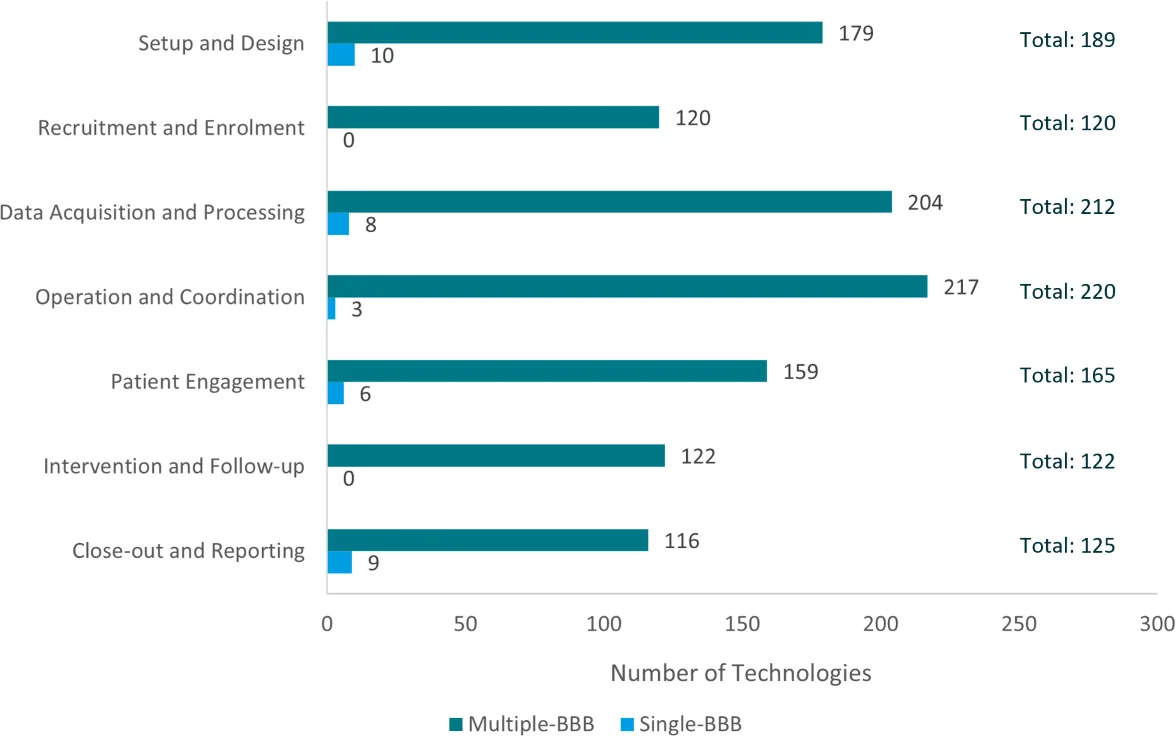
Number of technologies covering each basic building block (BBB) by single-BBB, multiple-BBB, and overall coverage.

**Figure 5.**
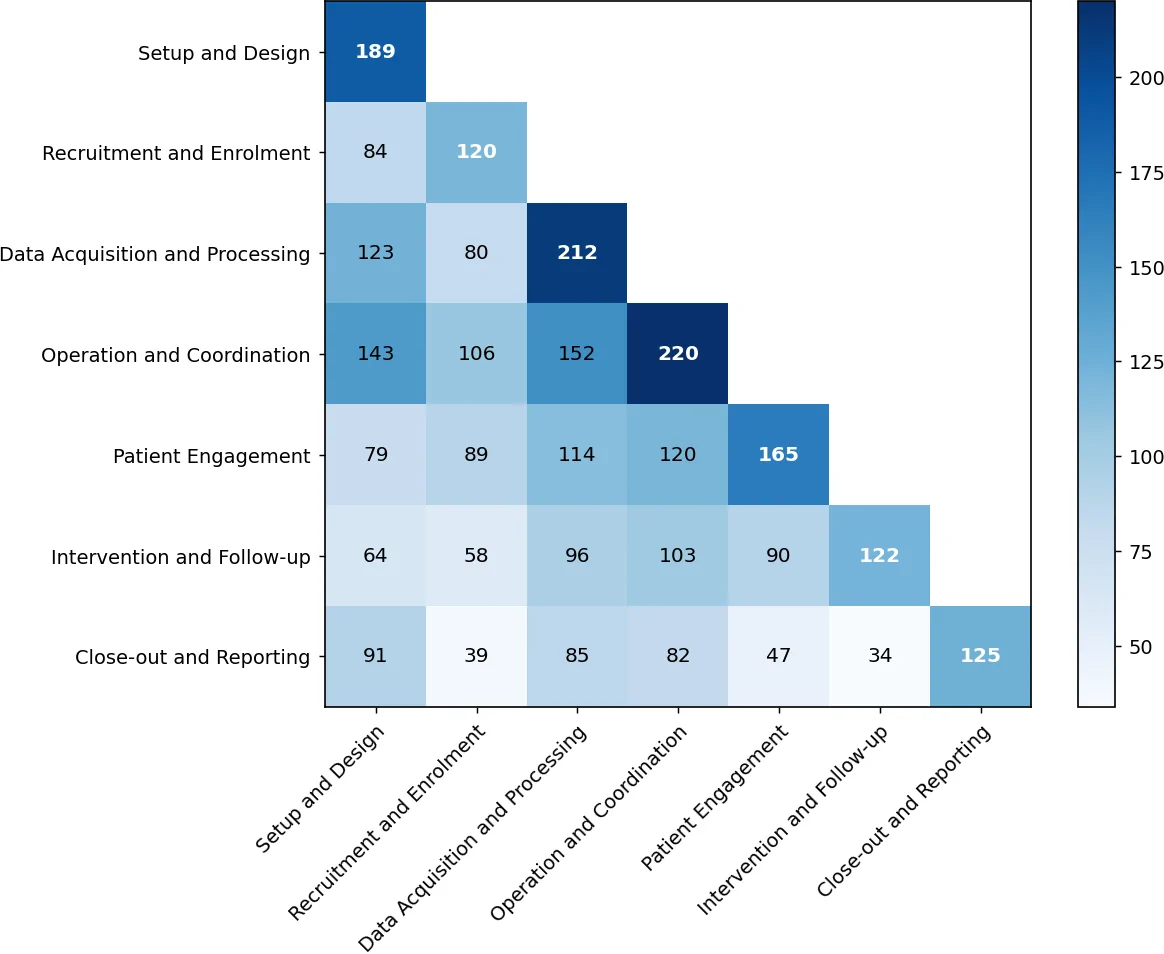
Co-occurrence heatmap of basic building block coverage among included technologies.

The strongest BBB pairings were data acquisition and processing with operation and coordination (n=152 technologies); setup and design with operation and coordination (n=143 technologies); and setup and design with data acquisition and processing (n=123 technologies). Operation and coordination also co-occurred frequently with patient engagement (n=120 technologies), while data acquisition and processing co-occurred with patient engagement in 114 technologies.

The most underrepresented pairings were intervention and follow-up with close-out and reporting (n=34 technologies); recruitment and enrollment with close-out and reporting (n=39 technologies); and patient engagement with close-out and reporting (n=47 technologies). These patterns suggest that planning, data, and operational functions were commonly combined, while later-stage close-out and reporting functions were less frequently combined with participant-facing or follow-up activities.

## Discussion

### Summary of Evidence

This scoping review provides an updated overview of 329 commercial technologies supporting decentralized and digitally enabled clinical trial operations. Using the BBB framework, the review maps how commercial technologies align with the clinical trial life cycle and identifies where functionality is concentrated or less frequently combined. Overall, the findings show that commercial technologies are available across all 7 BBBs, although their distribution and co-occurrence differ across operational areas.

The highest overall coverage was observed for operation and coordination, data acquisition and processing, and setup and design. This suggests strong commercial emphasis on operational management, data infrastructure, and trial planning functions. These areas are central to decentralized and hybrid trial implementation, where sponsors and investigators must maintain oversight, ensure reliable trial conduct, and manage data generated through digital and remote processes [31-33]. The prominence of data acquisition and processing is also consistent with the growing role of digital technologies, remote data acquisition, and Internet of Medical/Health Things approaches in decentralized clinical trials [32,34]. Setup and design coverage may reflect the continued importance of study planning, start-up, and protocol adaptation in the post-COVID-19 clinical trial environment [35].

Patient engagement also showed substantial coverage in the final catalog. This aligns with the increasing emphasis on participant-centric approaches in digitally enabled and decentralized research, including strategies intended to reduce participant burden, improve access, support retention, and maintain communication throughout trial participation [36-38]. However, patient engagement showed relatively weak co-occurrence with close-out and reporting, suggesting that participant-facing functions may remain less frequently combined with end-of-study reporting, archiving, and dissemination activities.

A major finding of the updated catalog was the predominance of multiple-BBB technologies. In the final dataset, 293 technologies were classified as multiple-BBB technologies, while 36 were classified as single-BBB technologies. This indicates that many commercial technologies are publicly described as supporting more than one area of the DCT operational life cycle. This finding should be interpreted cautiously, as it reflects publicly available vendor descriptions and BBB mapping rather than independent assessment of implementation depth or performance. Nevertheless, it is consistent with the growing role of platform-based and modular approaches in DCTs, where individual trial activities may correspond to specific platform modules and where integrated environments may help streamline trial processes [39,40].

The co-occurrence analysis showed that planning, data, and operational functions were commonly combined. The strongest BBB pairings were data acquisition and processing with operation and coordination; setup and design with operation and coordination; and setup and design with data acquisition and processing. These patterns suggest that commercial technologies often combine trial planning, data handling, and operational oversight. This is coherent with regulatory and implementation expectations for digital trial systems, where data integrity, validation, access management, audit trails, documentation, and oversight remain essential [31-33,41].

In contrast, the weakest co-occurrences involved close-out and reporting with intervention and follow-up, with recruitment and enrollment, and with patient engagement. This may indicate that close-out, archiving, reporting, and dissemination functions are less often combined with technologies used during participant-facing or active trial conduct phases. This finding highlights a potential for more connected technology support, particularly as decentralized and hybrid trials may require clearer continuity between data capture, operational oversight, participant interactions, and final reporting.

Compared with the previous 2020 technology scan [23], the current findings suggest a shift toward broader functional integration across the DCT technology landscape. The earlier scan identified the available technologies as relatively fragmented and siloed across trial activities, whereas the current review identified a predominance of technologies supporting multiple BBBs. This suggests that multifunctional and platform-based technologies are now more prominent, with some fragmentation still remaining in several BBB co-occurrences. The present review therefore refines the earlier conclusion by indicating broader functional coverage across the DCT life cycle while confirming that integration remains uneven across trial operations. These differences should be interpreted cautiously because the present review involved a more extensive technology-level reassessment and cross-BBB verification process than the earlier scan.

The present review also complements existing scoping reviews on digital technologies in clinical trials, digital therapeutics, and software as a medical device in DCTs, electronic health record-based recruitment support systems, and AI applications in clinical trials [17-20]. These reviews provide important insight into specific technology types, digital interventions, or selected trial operations. In contrast, the present study focuses on named commercial technologies and maps their operational coverage across the full DCT life cycle, providing a market-level catalog rather than a review of clinical study implementations alone.

The findings are also consistent with broader literature on the evolution of decentralized clinical trials. Previous work has described increasing adoption of hybrid and remote approaches across trial activities while also noting that some decentralized elements require further refinement and evaluation [42]. Recent work has also highlighted a gap between expectations and real-world DCT implementation, suggesting that DCT adoption remains uneven despite increasing interest [43]. Studies of decentralized research technology use and technology-enabled trial innovation similarly point to the need for infrastructure, operational readiness, and careful consideration of inclusivity, recruitment, engagement, and data quality [44,45]. By mapping 329 commercial technologies across all BBBs, this review provides a practical overview of available technologies and their potential role in supporting more connected DCT implementation.

Although regulatory, ethical, and implementation considerations were not extracted as formal data items, they remain important for interpreting the results. In DCT settings, sponsors and investigators must ensure participant safety, appropriate oversight, reliable implementation of decentralized elements, and secure remote data acquisition [31,32]. Systems handling electronic trial data should support validation, audit trails, user access management, data integrity, security, and documentation [33]. These considerations are consistent with Good Clinical Practice principles, which emphasize participant safety, data quality, and integrity in clinical trial design, conduct, and reporting [41]. When multiple technologies are combined across BBBs, trial teams should also consider interoperability, system integration, vendor oversight, accountability, participant burden, and escalation pathways.

In practice, the catalog can be used as an initial screening resource tool to identify technologies aligned with operational activities (BBBs) required for a specific decentralized or hybrid trial. BBB coverage should therefore be interpreted as documented functional alignment and not as evidence of regulatory compliance, clinical effectiveness, interoperability, or successful real-world implementation.

### Limitations

This study has limitations related to its design, data sources, classification process, and reliance on publicly available information. The BBB framework was useful for structuring the review, but clinical trial processes are highly interconnected and some activities may span more than 1 BBB. This introduced a degree of subjectivity in classification. Although expert review and consensus procedures were used to enhance consistency, formal interrater agreement statistics were not calculated.

The use of generative AI as an assistive tool during the June 2026 catalog update and verification step may have introduced a risk of incomplete or overstated suggestions. This risk was mitigated by treating all AI-assisted outputs only as draft suggestions, not as evidence sources, and by having human reviewers perform the actual evidence review, verification, and final BBB classification.

The review relied on publicly accessible English-language information, including vendor websites, product documentation, public reports, case studies, company announcements, and relevant academic literature. Paywalled sources, nonpublic vendor information, pricing, procurement data, vendor viability, standards compliance, and implementation performance were not systematically assessed. As a web-based commercial technology scan, identification may also have been influenced by search-engine indexing, ranking, and changes in online availability, while BBB classifications reflect publicly documented functionality rather than independent verification of regulatory compliance, interoperability, or real-world implementation performance. Therefore, some relevant technologies may have been missed if information was not publicly available, was not available in English, or required direct vendor engagement.

Although the search was rerun in June 2026 to improve currency, the commercial DCT technology landscape continues to evolve rapidly. New technologies may have emerged after the rerun, and existing technologies may have changed ownership, availability, or functionality after the final verification step. Periodic updates are therefore needed to maintain an accurate and useful catalog.

### Conclusions

This scoping review provides an updated mapping of 329 commercial technologies supporting decentralized and digitally enabled clinical trial operations. By combining a commercial technology scan with the BBB framework, the review offers a structured view of how available technologies align with the clinical trial life cycle and where functionality is concentrated or fragmented.

The findings can support sponsors, researchers, and trial teams in selecting and combining technologies for decentralized and hybrid trials. They also highlight the need for continued monitoring of the commercial DCT technology landscape, as technology availability, vendor positioning, and functional scope continue to evolve rapidly.

## Supplementary material

10.2196/86011 Multimedia Appendix 1 Full catalog of market scan technologies identified.

10.2196/86011 Multimedia Appendix 2 Standardized catalog verification prompt.

10.2196/86011 Checklist 1 PRISMA-ScR checklist.

10.2196/86011 Checklist 2 PRISMA-S checklist.

## Abbreviations

BBB: Basic Building Block
DCT: decentralized clinical trial
PRISMA-S: Preferred Reporting Items for Systematic Reviews and Meta-Analyses Literature Search Extension
PRISMA-ScR: Preferred Reporting Items for Systematic Reviews and Meta-Analyses Extension for Scoping Reviews
