# Supplementary material for: Quantifying the Digital Ecosystem of Commercial Technologies for Decentralized Clinical Trial Operations: Scoping Review
Source: JMIR Med Inform. 2026 Nov 12;14:e86011. doi: 10.2196/86011 (PMC13759182; doi:10.2196/86011)
Supplement: Supplementary file 1 [file medinform-v14-e86011-s002.docx]

**Standardized Catalogue Verification Prompt for the 2026 Update**

You are helping me update an existing catalogue of technologies for decentralised/digital clinical trial operations for the 2026 review cycle.

Your task is to generate structured draft suggestions for one existing catalogue row using only publicly available evidence. The output is intended to support human reviewer verification and should not be treated as final classification or as an evidence source.

# **Existing Catalogue Row**

Copy the values from the row below before running the prompt:

**Technology Name:** []
**Technology Vendor:** [FILL IN]
**Status:** [FILL IN]
**Link:** [FILL IN]
**Setup and Design:** [FILL IN: YES/NO]
**Recruitment and Enrolment:** [FILL IN: YES/NO]
**Data Acquisition and Processing:** [FILL IN: YES/NO]
**Operation and Coordination:** [FILL IN: YES/NO]
**Patient Engagement:** [FILL IN: YES/NO]
**Intervention and Follow-up:** [FILL IN: YES/NO]
**Close-out and Reporting:** [FILL IN: YES/NO]
**Number of BBBs Covered:** [FILL IN]
**Single-BBB / Multiple-BBB:** [FILL IN]
**Supported Activities Reported:** [FILL IN]

**Main Suggestion Tasks**

Check public sources and suggest whether:

1. The vendor/company still exists.
2. The specific Technology/product still appears active.
3. The existing link still works.
4. The Technology name, vendor name, or product page has changed.
5. The supported activities reported in the row are still accurate.
6. Each BBB currently marked **YES** is still supported by current public evidence.
7. Any BBB currently marked **NO** should now be changed to **YES** based on current public evidence.

Use this coding rule:

Mark a BBB as **YES** only if the technology clearly supports **at least one activity** listed under that BBB.
The technology does not need to support all activities within a BBB.
Do not infer BBB coverage from vague marketing language.

# **BBB Coding Guide**

## **1. Setup and Design**

Clinical trial planning, feasibility, and infrastructure preparation to allow successful trial execution.

Examples include protocol development; operational feasibility assessment; trial registration; informed consent form development; study branding; participant and site education strategy; site start-up; ethics and regulatory approvals; technology setup; investigational medicinal product supply; operational setup.

## **2. Recruitment and Enrolment**

Identifying, assessing, and enrolling potential participants.

Examples include participant outreach and pre-screening; participant education; informed consent; screening and randomisation; investigational medicinal product supply; patient technology enablement.

## **3. Data Acquisition and Processing**

Collection, storage, management, verification, transformation, and analysis of study data.

Examples include participant/device/sample data collection; study data management; data collection middleware; real-world data capture and management; clinical data repository management; eCRF and system query design; data reconciliation and query management; database lock; data transformation, standardisation, and analysis.

## **4. Operation and Coordination**

Operational conduct and coordination of the study.

Examples include clinical and performance monitoring; inspection and system approval facilitation; documentation management; home health and telemedicine visit management; regulatory and vendor management; safety data management; operational analytics; protocol and GCP deviation management; study oversight.

## **5. Patient Engagement**

Activities that support and maintain participant engagement throughout the trial.

Examples include patient engagement planning; social listening and patient landscape analysis; patient advocacy mapping; participant communication and updates; recruitment and retention incentives; concierge services or travel reimbursement; behavioural incentives; patient satisfaction surveys; advisory board engagement; patient–HCP interaction; direct messaging; patient community development.

## **6. Intervention and Follow-up**

Activities that collect study data and/or deliver interventions in accordance with the study protocol.

Examples include self-intervention and monitoring; home health visits; telemedicine visits.

## **7. Close-out and Reporting**

Activities to conclude the trial and report results.

Examples include decommissioning; archiving; study report production; clinical and operational results publication; scientific dissemination.

# **Review Status and Action Options**

**Suggested 2026 Review Status**

Suggest only one of the following for human reviewer consideration:

**Active**
The technology is still available/current and no major changes are needed.

**Updated**
The technology is still available/current, but the vendor name, product name, link, functionality, supported activities, or BBB coding needs updating.

**Inactive**
The technology appears discontinued, unavailable, or no longer has active public product evidence.

**Unclear**
The technology cannot be confidently verified from publicly available sources.

**Suggested 2026 Action**

Suggest only one of the following for human reviewer consideration:

**Keep**
Retain the entry without major changes.

**Update**
Retain the entry but revise information.

**Remove**
Remove from the updated catalogue.

**Discuss**
Needs team decision.

# **Required Output Format**

## Return the draft suggestions using the structure below.

## **1. Review Decision**

**2026 Review Status:
2026 Action:
Current Technology Name:
Current Vendor:
Current Best Link:
Old Link Works:** Yes / No / Unclear
**Evidence Source Used:
Date Checked:
Reviewer Initials:
Second Reviewer Initials:
Change Since Previous Version:** No change / Link updated / Vendor changed / Product renamed / Functionality changed / BBB coding changed / Unable to verify

Note: Use the date on which you completed the review. For Second Reviewer Initials, enter **N/A** if the second review has not yet been completed.

## **2. Evidence Summary**

Briefly summarise the public evidence found. Include what the source says the technology does. Do not invent missing information.

## **3. Supported Activities Confirmed**

List the supported activities that are clearly confirmed by public evidence.

## **4. BBB Coding Recommendation**

For each BBB, state whether to keep or change the current coding and explain why.

### **Setup and Design**

**Current coding:
Recommendation:** Keep as YES / Keep as NO / Change to YES / Change to NO / Discuss
**Rationale:**

### **Recruitment and Enrolment**

**Current coding:
Recommendation:** Keep as YES / Keep as NO / Change to YES / Change to NO / Discuss
**Rationale:**

### **Data Acquisition and Processing**

**Current coding:
Recommendation:** Keep as YES / Keep as NO / Change to YES / Change to NO / Discuss
**Rationale:**

### **Operation and Coordination**

**Current coding:
Recommendation:** Keep as YES / Keep as NO / Change to YES / Change to NO / Discuss
**Rationale:**

### **Patient Engagement**

**Current coding:
Recommendation:** Keep as YES / Keep as NO / Change to YES / Change to NO / Discuss
**Rationale:**

### **Intervention and Follow-up**

**Current coding:
Recommendation:** Keep as YES / Keep as NO / Change to YES / Change to NO / Discuss
**Rationale:**

### **Close-out and Reporting**

**Current coding:
Recommendation:** Keep as YES / Keep as NO / Change to YES / Change to NO / Discuss
**Rationale:**

## **5. Suggested Updated Coding for Human Review**

Return the suggested updated coding as YES/NO. Human reviewers will verify and finalize the coding.

**Setup and Design:
Recruitment and Enrolment:
Data Acquisition and Processing:
Operation and Coordination:
Patient Engagement:
Intervention and Follow-up:
Close-out and Reporting:**

## **6. Updated Derived Fields**

**Updated Number of BBBs Covered:
Updated Single-BBB / Multiple-BBB:**

## **7. Revised Supported Activities Reported**

Provide a concise revised text for the **Supported Activities Reported** column.

## **8. Draft Notes for Human Review**

**Draft Notes / Rationale:
Suggested Need for Team Discussion:** Yes / No
**If yes, specify what may need human discussion:**

# **Excel-Ready Fields**

At the end of the review, provide both formats below.

## **A. Two-Column Table**

| **Field name** | **Value** |
| --- | --- |
| 2026 Review Status |  |
| 2026 Action |  |
| Current Technology Name |  |
| Current Vendor |  |
| Current Best Link |  |
| Old Link Works |  |
| Evidence Source Used |  |
| Date Checked |  |
| Reviewer Initials |  |
| Second Reviewer Initials |  |
| Change Since Previous Version |  |
| Updated Setup and Design |  |
| Updated Recruitment and Enrolment |  |
| Updated Data Acquisition and Processing |  |
| Updated Operation and Coordination |  |
| Updated Patient Engagement |  |
| Updated Intervention and Follow-up |  |
| Updated Close-out and Reporting |  |
| Updated Number of BBBs Covered |  |
| Updated Single-BBB / Multiple-BBB |  |
| Revised Supported Activities Reported |  |
| Reviewer Notes / Rationale |  |
| Needs Team Discussion |  |
| Team Discussion Topic |  |

## **B. Comma-Separated Row**

Return a single comma-separated row of values only, in this exact order:

2026 Review Status
2026 Action
Current Technology Name
Current Vendor
Current Best Link
Old Link Works
Evidence Source Used
Date Checked
Reviewer Initials
Second Reviewer Initials
Change Since Previous Version
Updated Setup and Design
Updated Recruitment and Enrolment
Updated Data Acquisition and Processing
Updated Operation and Coordination
Updated Patient Engagement
Updated Intervention and Follow-up
Updated Close-out and Reporting
Updated Number of BBBs Covered
Updated Single-BBB / Multiple-BBB
Revised Supported Activities Reported
Reviewer Notes / Rationale
Needs Team Discussion
Team Discussion Topic

# **Important Rules**

Use only publicly available evidence.

Cite the public evidence used.

Where possible, use direct vendor/product pages as the primary evidence source. Secondary sources such as software directories, press releases, or articles can be used as supporting evidence, but final coding should be based on verifiable public information.

Do not invent missing information.

Do not classify a BBB as YES unless at least one listed activity under that BBB is clearly supported by the evidence.

If the product cannot be verified from public evidence, suggest Unclear and recommend Discuss or Remove for human reviewer consideration.

If the product is active but any information appears to have changed, suggest Updated and recommend Update for human reviewer consideration.

Provide enough detail for human reviewers to verify the sources, revise the output if needed, and transfer accepted information into the review spreadsheet.

Do not rely only on vague marketing language.

Do not use unsupported assumptions.

All outputs are suggestions only. Human reviewers must verify the public evidence, accept, revise, or reject the suggestions, and make the final decisions on inclusion, exclusion, supported activities, and BBB classification.
