# Supplementary material for: Quantifying the Digital Ecosystem of Commercial Technologies for Decentralized Clinical Trial Operations: Scoping Review
Source: JMIR Med Inform. 2026 Nov 12;14:e86011. doi: 10.2196/86011 (PMC13759182; doi:10.2196/86011)
Supplement: Supplementary file 3 [file medinform-v14-e86011-s004.docx]

**PRISMA-S Checklist**

| **Section/topic** | **#** | **Checklist item** | **Location(s) Reported** |
| --- | --- | --- | --- |
| **INFORMATION SOURCES AND METHODS** | | | |
| Database name | 1 | Name each individual database searched, stating the platform for each. | NA; no formal bibliographic database search was used.  Methods > Information Sources and Search, pages 8–10. |
| Multi-database searching | 2 | If databases were searched simultaneously on a single platform, state the name of the platform, listing all of the databases searched. | NA; no multi-database search platform was used,  Methods > Information Sources and Search, pages 8–10 |
| Study registries | 3 | List any study registries searched. | NA; study registries were not systematically searched or used as a primary search route.  Methods > Information Sources, page 8. |
| Online resources and browsing | 4 | Describe any online or print source purposefully searched or browsed (e.g., tables of contents, print conference proceedings, web sites), and how this was done. | Methods > Information Sources and Search, pages 8–10, Multimedia Appendix 1 |
| Citation searching | 5 | Indicate whether cited references or citing references were examined, and describe any methods used for locating cited/citing references (e.g., browsing reference lists, using a citation index, setting up email alerts for references citing included studies). | Not systematically conducted; relevant academic literature and prior work were used for contextualization.  Methods > Information Sources, page 8. |
| Contacts | 6 | Indicate whether additional studies or data were sought by contacting authors, experts, manufacturers, or others. | NA; no systematic author, expert, or vendor contact was conducted.  Methods > Information Sources and Limitations, pages 8 and 21. |
| Other methods | 7 | Describe any additional information sources or search methods used. | Methods > Protocol and Registration; Information Sources; Search; Selection of Sources of Evidence, pages 7–11. |
| **SEARCH STRATEGIES** | | | |
| Full search strategies | 8 | Include the search strategies for each database and information source, copied and pasted exactly as run. | Because no formal bibliographic database search was used, BBB-aligned web-search terms are reported instead of database-specific search strings.  Methods > Search; Table 2, pages 9–10. |
| Limits and restrictions | 9 | Specify that no limits were used, or describe any limits or restrictions applied to a search (e.g., date or time period, language, study design) and provide justification for their use. | Methods > Eligibility Criteria; Information Sources; Search; Limitations, pages 7–10 and 20. |
| Search filters | 10 | Indicate whether published search filters were used (as originally designed or modified), and if so, cite the filter(s) used. | NA, no published search filters were used. Methods > Search, page 9. |
| Prior work | 11 | Indicate when search strategies from other literature reviews were adapted or reused for a substantive part or all of the search, citing the previous review(s). | NA, no search strategy from another literature review was directly adapted or reused, The previous 2020 Trials@Home technology scan was used as the baseline dataset for reassessment, while the updated search used the BBB-aligned web-based strategy described.  Methods > Protocol and Registration; Information Sources; Selection of Sources of Evidence, pages 7–11. |
| Updates | 12 | Report the methods used to update the search(es) (e.g., rerunning searches, email alerts). | Methods > Information Sources; Search; Selection of Sources of Evidence; Data Charting Process, pages 8–12. |
| Dates of searches | 13 | For each search strategy, provide the date when the last search occurred. | Abstract and Methods > Information Sources and Search, pages 1–2 and 8–10, Multimedia Appendix 1. |
| **PEER REVIEW** | | | |
| Peer review | 14 | Describe any search peer review process. | Methods > Selection of Sources of Evidence; Data Charting Process, pages 10–12. |
| **MANAGING RECORDS** | | | |
| Total Records | 15 | Document the total number of records identified from each database and other information sources. | Results > Selection of Sources of Evidence; Figure 3; Multimedia Appendix 1, page 14. |
| Deduplication | 16 | Describe the processes and any software used to deduplicate records from multiple database searches and other information sources. | Duplicate entries were removed manually through cross-checking reviewer outputs and source lists; no specialized deduplication software was used.,  Methods > Selection of Sources of Evidence; Results > Selection of Sources of Evidence, pages 10–11 and 14. |
|  |  |  |  |
| PRISMA-S: An Extension to the PRISMA Statement for Reporting Literature Searches in Systematic Reviews | | |  |
| Rethlefsen ML, Kirtley S, Waffenschmidt S, Ayala AP, Moher D, Page MJ, Koffel JB, PRISMA-S Group. | | |  |
| Last updated February 27, 2020. | |  |  |
